# Recommended core outcome instruments for health‐related quality of life, long‐term control and itch intensity in atopic eczema trials: results of the HOME VII consensus meeting*

**DOI:** 10.1111/bjd.19751

**Published:** 2021-02-08

**Authors:** K.S. Thomas, C.A. Apfelbacher, J.R. Chalmers, E. Simpson, P.I. Spuls, L.A.A. Gerbens, H.C. Williams, J. Schmitt, M. Gabes, L. Howells, B.L. Stuart, E. Grinich, T. Pawlitschek, T. Burton, L. Howie, A. Gadkari, L. Eckert, T. Ebata, M. Boers, H. Saeki, T. Nakahara, N. Katoh

**Affiliations:** ^1^ Centre of Evidence Based Dermatology School of Medicine Nottingham UK; ^2^ Institute of Social Medicine and Health Systems Research (ISMHSR) Otto von Guericke University Magdeburg Magdeburg Germany; ^3^ Department of Dermatology Oregon Health & Science University Portland OR USA; ^4^ Department of Dermatology Amsterdam Public Health, Infection and Immunity, Amsterdam UMC University of Amsterdam Amsterdam the Netherlands; ^5^ Center for Evidence‐based Healthcare Medical Faculty Carl Gustav Carus Dresden Germany; ^6^ Medical Sociology Department of Epidemiology and Preventive Medicine University of Regensburg Regensburg Germany; ^7^ Primary Care, Population Sciences and Medical Education Faculty of Medicine University of Southampton Southampton UK; ^8^ School of Medicine (Department of Dermatology) Oregon Health & Science University Portland OR USA; ^9^ Patient representative (independent) Nottingham UK; ^10^ Global Parents for Eczema Research Brisbane Australia; ^11^ Health Economics and Outcomes Research Boehringer Ingelheim Inc. Ingelheim, Rheinland‐Pfalz Germany; ^12^ Global Dupixent Business Partner sanofi GHEVA 1 av. Pierre Brossolette Chilly‐Mazarin 91380 France; ^13^ Chitofuna Dermatology Clinic Tokyo Japan; ^14^ Department of Epidemiology and Data Science Amsterdam Rheumatology and Immunology Center Amsterdam UMC, Vrije Universiteit Amsterdam the Netherlands; ^15^ Department of Dermatology Nippon Medical School 1‐1‐5 Sendagi, Bunkyo‐ku Tokyo Japan; ^16^ Department of Dermatology Graduate School of Medical Sciences Kyushu University Fukuoka Japan; ^17^ Department of Dermatology Graduate School of Medical Science Kyoto Prefectural University of Medicine Kyoto Japan

## Abstract

**Background:**

The Harmonising Outcome Measures for Eczema (HOME) initiative has established a core outcome set of domains for atopic eczema (AE) clinical trials. Previous consensus meetings have agreed on preferred instruments for clinician‐reported signs (Eczema Area and Severity Index, EASI) and patient‐reported symptoms (Patient‐Oriented Eczema Measure, POEM). This paper reports consensus decisions from the HOME VII meeting.

**Objectives:**

To complete the core outcome set for AE by agreeing on core outcome instruments for the domains of quality of life (QoL), long‐term control and itch intensity.

**Methods:**

A face‐to‐face consensus meeting was held in Tokyo, Japan (8–10 April 2019) including 75 participants (49 healthcare professionals/methodologists, 14 patients, 12 industry representatives) from 16 countries. Consensus decisions were made by presentations of evidence, followed by whole and small group discussions and anonymous voting using predefined consensus rules.

**Results:**

It was agreed by consensus that QoL should be measured using the Dermatology Life Quality Index (DLQI) for adults, the Children’s Dermatology Life Quality Index (CDLQI) for children and the Infant’s Dermatology Quality of Life Index (IDQoL) for infants. For long‐term control, the Recap of Atopic Eczema (RECAP) instrument or the Atopic Dermatitis Control Test (ADCT) should be used. Consensus was not reached over the frequency of data collection for long‐term control. The peak itch numerical rating scale (NRS)‐11 past 24 h was recommended as an additional instrument for the symptom domain in trials of older children and adults. Agreement was reached that all core outcome instruments should be captured at baseline and at the time of primary outcome assessment as a minimum.

**Conclusions:**

For now, the core outcome set for clinical trials in AE is complete. The specified domains and instruments should be used in all new clinical trials and systematic reviews of eczema treatments.

## Abstract 2

What is already known about this topic?

- Core outcomes sets improve the design and reporting of clinical trials, reduce selective outcome reporting bias and facilitate meta‐analysis of results in systematic reviews.
- The HOME core outcome set for eczema recommends the inclusion of four core domains in all atopic eczema trials: clinician‐reported signs, patient‐reported symptoms, health‐related quality of life (HrQoL) and long‐term control.
- Clinician‐reported signs should be captured using the Eczema Area and Severity Index (EASI) and patient‐reported symptoms using the Patient‐Oriented Eczema Measure (POEM).

What does this study add?

- The HOME core outcome set is now complete and recommended core outcome instruments have been agreed on for all four domains.
- Core outcome instruments for HrQoL: Dermatology Life Quality Index (DLQI) for adults, Children’s Dermatology Life Quality Index (CDLQI) for children and Infant’s Dermatology Quality of Life Index (IDQoL) for infants.
- Core outcome instruments for long‐term control: either the Recap of Atopic Eczema (RECAP) or the Atopic Dermatitis Control Test (ADCT).
- In addition, itch intensity should be measured using the peak NRS‐11 past 24 h for trials including older children and adults.

What are the clinical implications of this work?

- If all future trials of eczema treatments include the HOME core outcome instruments, then trial results will be more readily incorporated into meta‐analyses in systematic reviews and clinical care will be informed by the best available evidence.

**Linked Comment:** D.F. Murrell and C.F. Paul. *Br J Dermatol* 2021; **185**:13–14.

Core outcome sets are considered an essential factor for progressing evidence‐based medicine by permitting studies to be compared and combined in a meaningful way in future systematic reviews and meta‐analyses.^1^ Core outcome sets also ensure the inclusion and reporting of valid endpoints that are meaningful to patients. The Harmonising Outcomes Measures for Eczema (HOME) initiative has proposed four core domains to be captured in all clinical trials of atopic eczema (AE, syn. atopic dermatitis, eczema) treatments: clinician‐reported signs, patient‐reported symptoms, health‐related quality of life (HrQoL) and long‐term control.^2^ The outcome measurement instruments recommended for capturing signs and symptoms are the Eczema Area and Severity Index (EASI) and Patient‐Oriented Eczema Measure (POEM), respectively.^2^, ^3^, ^4^

HrQoL has been discussed at HOME consensus meetings previously, for adults (HOME IV) and children (HOME V),^5^, ^6^ but consensus had not been reached over the preferred instruments to recommend for the core outcome set.

The domain of long‐term control was the main topic of discussion at the HOME V meeting in Nantes in 2017, where it was agreed that long‐term control should be captured by a combination of repeated measurement of existing core domains (signs, symptoms and HrQoL), plus a patient global assessment of control. It was recommended that these aspects should be measured repeatedly during trials wherever possible, and that further research was required to agree on a preferred instrument for capturing eczema control, as well as the optimum frequency and timing of assessments.^6^

For the symptoms domain, itch intensity had been proposed previously as an important addition to the POEM instrument, which captures frequency of symptoms including itch, but does not quantify itch severity.^5^

Achieving consensus over the preferred instruments to complete the HOME core outcome set for AE trials was the focus of discussions at a 3‐day consensus meeting held in Tokyo, Japan (8–10 April 2019) – HOME VII. This report outlines a summary of the consensus recommendations from the HOME VII meeting.

## Methods

### Organization of consensus meeting and consensus rules

Methods used to select outcome instruments for inclusion in the core outcome set were guided by the HOME methodological Roadmap^7^ and internationally agreed guidance for selecting outcome instruments.^8^ The latter recommends four key stages to the selection of core outcome instruments: conceptual considerations, identification of existing instruments, quality assessment of instruments, and generic aspects (e.g. minimum criteria for inclusion should be content validity, internal consistency if relevant and feasibility). Decisions during the meeting were informed by up‐to‐date systematic reviews of validation studies for relevant outcome measurement instruments.

Seventy‐five delegates attended the meeting, plus five observers/translators including 43 (57%) healthcare professionals, 14 (19%) patients/representatives, six (8%) methodologists and 12 (16%) industry representatives (Table S1; see Supporting Information). The meeting included a mixture of presentations, whole and small group discussions, and anonymous voting using electronic keypads.

Discussions focused on establishing agreement over: (i) preferred instruments for measuring HrQoL in adults and children; (ii) long‐term control in adults and children; and (iii) itch intensity in adults (as a proposed additional instrument to capture an essential missing element of the symptoms domain). In addition, discussion of the recommended frequency of data collection and timing of assessments was debated.

Small group discussions were used to evaluate content validity and feasibility, using summary cards as prompts to generate discussion and to rank instrument options. Anonymized electronic voting was employed to reach consensus decisions. As with previous HOME meetings, consensus was reached on a statement when fewer than 30% of voters disagreed. Individuals involved in the development of specific instruments were excluded from the vote to avoid conflicts of interest.

Full details of the methods used are provided in the published meeting report.

## Results

### Quality of Life domain

#### Domain definition

Quality of life is a complex, often multidimensional construct that is usually measured by multi‐item questionnaires. At the HOME IV meeting, the HOME initiative voted that psychological functioning, social functioning and physical functioning are essential subdomains for the construct QoL and that there are no other essential subdomains.^5^ Available instruments for AE differ according to age group: different instruments exist for infants, children and adults. There is not one QoL instrument for AE that can be used across all ages.

#### Evidence provided at the meeting

An updated systematic review of the measurement properties of QoL outcome measures for infants, children and adults with AE was presented.^9^ According to this systematic review, none of the existing patient‐reported outcome measures for QoL could be recommended for use. In addition, a newly developed short‐form of the Childhood Atopic Dermatitis Impact Scale (CADIS) was presented.^10^ This scale showed excellent internal consistency, test–retest reliability, construct validity and responsiveness and was considered to be a more feasible scale than the original 45‐item CADIS.^11^

#### Small group discussions and feedback

Content validity is recommended as a primary consideration when selecting a core outcome instrument.^8^ Small group discussions therefore focused on rating the content validity of each of nine candidate QoL instruments.^12^ Each group consisted of a mixture of clinicians, patients, industry representatives and methodologists, and discussions were guided by a facilitator (a member of the HOME executive committee). Each group discussed either one or two instruments. The COnsensus‐based Standards for the selection of health Measurement INstruments (COSMIN) criteria^13^ on relevance, comprehensiveness and comprehensibility were used to determine the overall content validity rating per instrument. The facilitator of each group presented their findings and final ratings in plenary (Table 1).

**Table 1:** Summary of small group content validity rating on quality of life candidate instruments

	Group 1 *n* = 10		Group 2 *n* = 10		Group 3 *n* = 11	Group 4 *n* = 11	Group 5 *n* = 8		Group 6 *n* = 12
	IDQoL	CDLQI	CADIS (long‐form)	CADIS (short‐form)	DISABKIDS (proxy‐ and self‐reported)	InToDermQoL	DLQI	Skindex‐16	ABS‐A
Content validity rating	+	+	+	±	–	–	±	±	–

IDQoL, Infant’s Dermatitis Quality of Life Index; CDLQI, Children’s Dermatology Life Quality Index; CADIS, Childhood Atopic Dermatitis Impact Scale; DISABKIDS, the European DISABKIDS Group questionnaire; InToDermQoL, Infants and Toddlers Dermatology Quality of Life; DLQI, Dermatology Life Quality Index; ABS‐A, Atopic Dermatitis Burden Scale for Adults; +, sufficient; –, insufficient; ±, inconsistent

#### Consensus vote

Whether an instrument could be recommended as the core outcome instrument for QoL was voted on for each age group. Seventy‐two attendees took part in the consensus voting (five conflicted attendees abstained). The Infant’s Dermatitis Quality of Life Index (IDQoL) was voted as the recommended instrument for infants [8 of 70 (11%) disagreed] and the Children’s Dermatology Life Quality Index (CDLQI) for children [6 of 69 (9%) disagreed]. For adults, both the Dermatology Life Quality Index (DLQI) and Skindex were voted as potentially suitable candidate instruments for the core set [9 of 72 (13%) and 16 of 70 (23%) disagreed, respectively], but following further discussion and a second consensus vote, the DLQI was chosen as the preferred instrument for adults [11 of 72 (15%) disagreed].

In conclusion, the family of instruments, IDQoL, CDLQI and DLQI, which are the most frequently used instruments in the literature, were recommended as the core outcome instruments for the QoL domain in AE clinical trials.

### Long‐term control domain

#### Domain definition

**Figure 1:**
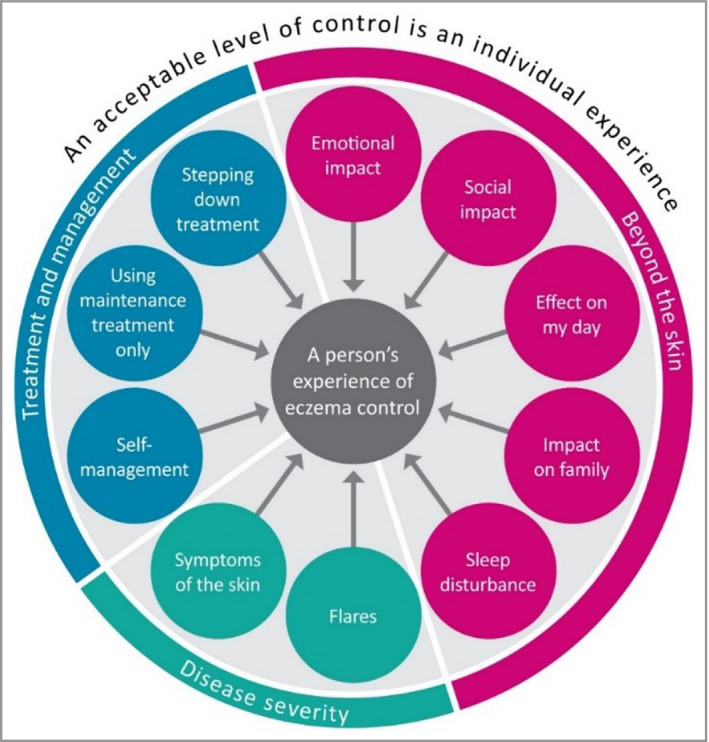
Conceptual framework for eczema control (reproduced with permission from Howells *et al*.^14^).

A conceptual model for the construct of ‘long‐term control’ was presented and debated (Figure 1, reproduced with permission).^14^ This model was based on mixed‐methods research and stakeholder opinion to define the construct of eczema control.^6^, ^15^, ^16^, ^17^

The HOME domain of ‘long‐term control’ was clarified as being ‘eczema control’ measured repeatedly over time. This domain is relevant to adults and children with eczema, and to people with all severities of disease.

#### Instruments considered

In preparation for the meeting, five possible outcome measurement instruments to capture ‘patient‐reported eczema control’ were identified. Four were multi‐item instruments [Atopic Dermatitis Control Test (ADCT),^18^ Recap of Atopic Eczema (RECAP),^14^ Patient Benefit Index (PBIv2·0)^19^, ^20^, ^21^ and Atopic Dermatitis Score 7 (ADS7)^22^] and one was a single‐item global eczema severity instrument Patient Global Assessment (PtGA).^23^, ^24^ The ADCT and RECAP instruments were specifically developed to measure ‘eczema control’, PBI is a measure of patient‐related benefit following dermatology treatment (but is not eczema‐specific) and ADS7 assesses eczema symptoms and burden of disease. These instruments were identified through a systematic review of the literature and via a survey of the HOME membership to identify newly developed and unpublished instruments.

Quality assessment of the instruments was conducted prior to the meeting according to COSMIN methodology.^13^ Content validity for capturing eczema control was insufficient for the ADS7 instrument and so this instrument was not considered further. All instruments were felt to be feasible to include in the core outcome set. Three instruments (ADCT, RECAP and PtGA) had evidence of ‘sufficient’ content validity, PBI had ‘inconsistent’ evidence of content validity (manuscript in preparation).

During the consensus meeting, individual instruments were discussed and ranked in six small groups with a variety of stakeholders in each group. The results of these small group discussions are shown in Table 2. The majority of groups preferred a single‐item ‘global eczema control’ instrument, but five of the six groups felt that the existing validated PtGA instrument was not suitable for this purpose as the response options of mild, moderate or severe disease were not seen as being appropriate to the construct being measured, and there was no clear option. It was also noted that the domain measured by the PtGA was disease severity and not disease ‘control’, per se. Development and validation of a single‐item global eczema control instrument is therefore required. All groups agreed that the PBI instrument was not suitable as an ‘eczema control’ measure because it asks about needs and benefits of treatment rather than ‘eczema control’ and so was rapidly rejected by most groups. Of the remaining two multi‐item control instruments, both RECAP and ADCT were judged to be of high quality and similar in content, making it difficult to choose one over the other.

**Table 2:** Summary of small group feedback on long‐term control outcome instruments

	Group 1	Group 2	Group 3	Group 4	Group 5	Group 6
Single‐item / multi‐item preferred	50/50 split	N/A	Single	Single	Single	Multi
*Single‐item instruments*						
Existing validated PtGA suitable?	No	No	No	No	Yes	No
Development / validation of a new single‐item instrument required?	Yes	Yes	Yes	Yes	Yes	Yes
*Multi‐item instruments*						
1st‐choice instrument	RECAP	RECAP	RECAP	RECAP	N/A	RECAP
2nd‐choice instrument	ADCT	ADCT	ADCT	ADCT	N/A	ADCT
PBIv2·0 and ADS7	NP	NP	NP	NP	NP	NP

ADCT, Atopic Dermatitis Control Test instrument; ADS7, Atopic Dermatitis Score 7; PBI, Patient Benefit Index; PtGA, Patient Global Assessment instrument; RECAP, Recap of eczema control instrument; N/A, not applicable or not ranked; NP, not preferred

#### Consensus voting

Following whole group discussion and indicative voting, 54 attendees took part in the consensus voting (12 conflicted attendees abstained). ADCT and RECAP were both voted as potential candidate instruments for the core outcome set [15 of 54 (27·8%) and 16 of 54 (29·6%), respectively, disagreed with their inclusion].

It was also recommended that further research should be conducted to develop a single‐item global control instrument that could be evaluated at future HOME meetings.

As ADCT and RECAP were both newly developed (and very similar) instruments, with limited validation data to inform a choice between the two, it was agreed that both should be recommended for use as core outcome instruments to measure long‐term control for the time being [12 of 53 (23%) disagreed].

No consensus was reached over the preferred frequency of data collection for the domain of long‐term control. This will be discussed at future HOME meetings.

### Itch intensity (additional symptom instrument)

#### Domain definition

Itch intensity was defined as intensity measured by a single‐item instrument, such as a numerical rating scale (NRS), verbal rating scale (VRS) or visual analogue scale (VAS), measured over a specific period of time, e.g. the past 24 h or past week. This subdomain is relevant to adults and children with eczema, with all severities of disease.

#### Instruments considered

Evidence was presented on outcome measurement instruments of itch intensity regarding instrument properties and quality of the evidence on measurement properties. Instruments were identified through two systematic reviews^25^, ^26^ and an additional search update. Further, a pre‐meeting survey of the HOME membership (106 respondents: 50 clinicians, 33 patients or patient representatives, 14 industry representatives, seven methodologists, two not stated) identified the preferences over response options (NRS, VRS, VAS), type of intensity (peak itch, average itch) and recall period (past 24 h, past week) of itch intensity instruments (Table 3).

**Table 3:** Results of pre‐meeting survey of HOME membership regarding itch preferences

*Would you prefer response options using:*	
Numerical rating scale (NRS)	50 (47%)
Verbal rating scale (VRS)	22 (21%)
Visual analogue scale (VAS)	24 (23%)
Unsure	7 (7%)
Not stated	3 (3%)
*Would you prefer itch intensity to be measured using:*	
Average itch	45 (42·5%)
Peak itch	45 (42·5%)
Unsure	9 (8%)
Not stated	7 (7%)
*Which of the following timescales do you consider to be the most appropriate for capturing itch intensity?*	
Past 24 h	41 (39%)
Past week	48 (45%)
None of these	9 (8%)
Not stated	8 (7·5%)

A systematic review of measurement properties for patient‐reported outcome measures of itch intensity included 23 studies on 23 instruments (search 1965–2015).^25^ Preliminary results of an update to this review were presented at the HOME meeting and included 14 extra studies, resulting in a total number of 32 instruments for consideration (search 2015–2019) (publication in preparation). In addition, the systematic review of measurement properties of eczema symptoms, including itch intensity, was presented (search 1965–2015).^26^ Eighteen instruments were included. An update revealed only one new paper in eczema concerning itch intensity.^27^

Based on the quality assessment of the instruments and studies using COSMIN methodology, the following single‐item instruments were considered: (i) investigated in patients with itch: NRS‐6 (recall period not specified), peak NRS‐11 past 24 h, verbal NRS (VNRS)‐4 (recall period variable), VAS (horizontal) (recall period variable)^25^ and (ii) investigated in adult patients with eczema: VAS (horizontal) past 24 h,^28^ VRS‐5 past 24 h^28^ and NRS‐11 past 24 h.^27^

Only three instruments were validated for eczema in adults, and only for the recall period of the past 24 h. The VAS and VRS past 24 h^28^ had limited evidence for construct validity. The peak NRS‐11 past 24 h^27^ has been validated for several measurement properties (i.e. content validity, test–retest reliability, discriminating/known groups validity, sensitivity to change, construct validity). This study indicated that peak itch was easier to remember and to rate compared with average itch; also, peak interpretation was more precise and consistent.

#### Consensus voting

After whole group discussions, 63 attendees took part in the consensus voting. Only five of 63 (8%) disagreed with the statement that itch intensity should be included in the symptoms domain and reported in addition to POEM. All industry representatives refrained from the remainder of the voting due to perceived or potential conflicts of interest with the instruments. It was agreed that peak itch should be measured rather than average itch; only three of 50 (6%) disagreed with including peak itch, whereas 34 of 50 (68%) disagreed with including average itch. The recall period ‘past 24 hours’ for capturing peak itch intensity was preferred with only four of 50 (8%) disagreeing with this timescale compared with more than half disagreeing with using the recall period ‘past week’ (28 of 50, 56%).

Consensus was reached to include the peak NRS‐11 past 24 h^27^ as the core outcome instrument for measuring the subdomain of itch intensity in adults [4 of 48 (8%) disagreed]. In this instrument, peak itch is measured by the following question: ‘On a scale of 0 to 10, with 0 being “no itch” and 10 being “worst itch imaginable”, how would you rate your itch at the worst moment during the previous 24 hours?’^27^

This recommendation was made only for adults, as there was no validation data available for younger people. However, the group suggested using the instrument for anyone who can self‐report. It is not considered appropriate as a proxy measure.

In the future, further validation data on NRS‐11, including in younger people, will be investigated.

### Frequency of data collection

Discussion over the optimal timing and frequency of data collection for the core outcome instruments was held. Discussions were informed by a pre‐meeting survey, which scoped the views of the wider HOME membership over the preferred timing and frequency of data collection for the core outcome set instruments.

It was agreed by consensus that core outcomes should be collected, as a minimum, at baseline and at the time of the primary outcome assessment, which is usually equivalent to end of treatment in trials [none of 72 (0%) disagreed]. No agreement was reached as to the preferred timing of outcome assessments at other timepoints but it was agreed that recommendations (not mandatory) on timepoints for the core outcomes set should be made as part of future HOME activities [nine of 72 (13%) disagreed].

## Discussion

During this 3‐day HOME consensus meeting, agreement was reached over the preferred instruments for capturing HrQoL, long‐term control and itch intensity. It was also agreed that outcomes should be recorded as a minimum at baseline and at the time of the primary outcome assessment. These recommendations build on the previously agreed core outcome recommendations and completes the HOME core outcome set for AE.

For HrQoL, the preferred core outcome instruments are IDQoL, CDLQI and DLQI for infants, children and adults, respectively.^29^, ^30^, ^31^ This recommendation is welcomed as the inclusion of HrQoL assessment in clinical trials has been limited by lack of recommended outcome instruments.

For long‐term control, the RECAP and ADCT outcome instruments^14^, ^32^ are recommended and for the symptoms domain it is recommended that itch intensity be collected for adults and older children using a 0–10 NRS instrument of peak itch in the last 24 h, in addition to POEM.

Future research and consensus discussions are required to evaluate new QoL instruments as they become available, to explore the use of Patient‐Reported Outcomes Measurement Information System (PROMIS) instruments^33^ for assessing different aspects of QoL, to develop and validate a single‐item global assessment of eczema control, to further validate the RECAP and ADCT instruments and to establish the optimum frequency and timing of outcome assessments to facilitate meta‐analysis of trial findings.

In considering strengths and limitations, these consensus decisions were conducted over 3 days during a face‐to‐face meeting led by an Independent Chair who had 20+ years of experience of developing core outcomes sets (M.B.). Decisions were made based on the best available evidence, including the results of systematic reviews, surveys of the HOME membership, and international online patient discussion groups conducted prior to the consensus meeting. Our methods were informed by guidance from Outcome Measures in Rheumatology (OMERACT),^34^ COMET,^35^ COSMIN^8^ and the HOME Roadmap.^7^

Because patients and patient representatives represented just 19% of attendees at the meeting, it is possible that some felt it difficult to express their thoughts during the meeting. We strengthened the patient voice by holding a separate session for patients prior to the main meeting and by using small group consensus methods to inform decisions, thus enabling patients to engage more fully. We also had translators working with patients attending the meeting who were unable to speak English.

In conclusion, for now, the core outcome set for clinical trials in AE is complete and the specified outcome domains and outcome measurement instruments should be used in all new clinical trials and systematic reviews of AE treatments. Ongoing efforts are required to ensure global uptake of the agreed core outcome instruments. Regulatory authorities, journal editors and funders can play a key role in supporting implementation.

## Author Contribution

**Kim S Thomas:** Conceptualization (equal); Data curation (equal); Methodology (equal); Writing‐original draft (lead); Writing‐review & editing (equal). **Christian Apfelbacher:** Conceptualization (equal); Data curation (equal); Methodology (equal); Writing‐original draft (equal); Writing‐review & editing (equal). **Joanne Rachel Chalmers:** Conceptualization (equal); Methodology (equal); Writing‐review & editing (equal). **Eric Lawrence Simpson:** Conceptualization (equal); Methodology (equal); Writing‐review & editing (equal). **Phyllis Spuls:** Conceptualization (equal); Methodology (equal); Writing‐original draft (supporting); Writing‐review & editing (equal). **Louise A.A. Gerbens:** Conceptualization (equal); Methodology (equal); Writing‐original draft (supporting); Writing‐review & editing (equal). **Hywel Williams:** Conceptualization (equal); Methodology (equal); Writing‐review & editing (equal). **Jochen Schmitt:** Conceptualization (equal); Methodology (equal); Writing‐review & editing (equal). **Michael Gabes:** Conceptualization (equal); Methodology (equal); Writing‐original draft (supporting); Writing‐review & editing (equal). **Laura Howells:** Conceptualization (equal); Methodology (equal); Writing‐review & editing (equal). **Beth Stuart:** Conceptualization (equal); Methodology (equal); Writing‐review & editing (equal). **Erin E Grinich:** Conceptualization (equal); Methodology (equal); Writing‐review & editing (equal). **Traci Pawlitschek:** Conceptualization (equal); Methodology (equal); Writing‐review & editing (equal). **Tim Burton:** Conceptualization (equal); Methodology (equal); Writing‐review & editing (equal). **Lynita Howie:** Conceptualization (equal); Methodology (equal); Writing‐review & editing (equal). **Abhijit Gadkari :** Conceptualization (equal); Methodology (equal); Writing‐review & editing (equal). **Laurent Eckert:** Conceptualization (equal); Methodology (equal); Writing‐review & editing (equal). **Toshiya Ebata:** Conceptualization (equal); Methodology (equal); Writing‐review & editing (equal). **Maarten Boers:** Conceptualization (equal); Methodology (equal); Writing‐review & editing (equal). **Hidehisa Saeki:** Conceptualization (equal); Methodology (equal); Writing‐review & editing (equal). **Takeshi Nakahara:** Conceptualization (equal); Methodology (equal); Writing‐review & editing (equal). **Norito Katoh:** Conceptualization (equal); Methodology (equal); Writing‐review & editing (equal).

## Supporting information

**Table S1** HOME VII participants who contributed to the consensus meeting and voting (8–10 April 2019).
**Powerpoint S1** Journal Club Slide Set.

## Appendix 1 Funding sources

Delegates were charged a fee as follows: patients free of charge, nonindustry £100, industry £500. All delegates covered their own travel and accommodation expenses with the exception of patients and representatives from patient groups, whose costs were met by either a stipend from the local organizing committee, their national eczema patient association or by a participating HOME member. H.C.W. supported the fees, travel and accommodation costs for M.B. (OMERACT) to attend as the independent moderator, using funds from his National Institute for Health Research Senior Investigator award. Contributions by L. Howells were supported by a PhD studentship from the British Skin Foundation (Ref: 8016). Grants to support the meeting were provided by the Japanese Dermatological Association, Kyoto Prefectural University of Medicine and Kyushu University, The Uehara Memorial Foundation, The Nakatomi Foundation and Lydia O’Leary Memorial Pias Dermatological Foundation.
