# Supplementary material for: Recommended core outcome instruments for health‐related quality of life, long‐term control and itch intensity in atopic eczema trials: results of the HOME VII consensus meeting
Source: Br J Dermatol. 2021 Feb 8;185(1):139–46. doi: 10.1111/bjd.19751 (PMC8359383; doi:10.1111/bjd.19751)
Supplement: Supplementary file 1 — Table S1 HOME VII participants who contributed to the consensus meeting and voting (8–10 April 2019). Powerpoint S1 Journal Club Slide Set. [file BJD-185-139-s001.docx]

# Table S1: HOME VII participants who contributed to the consensus meeting and voting (8–10 April 2019)

Maarten Boers: independent facilitator

| 1. Abhijit Gadkari | Pharmaceutical Industry |
| --- | --- |
| 1. Akane Yasui | Patient / patient representative |
| 1. Åke Svensson | Clinician |
| 1. Alison Sears | Clinician |
| 1. Alix Bullock | Patient / patient representative |
| 1. Amy DeLozier | Pharmaceutical Industry |
| 1. Andreas Wollenberg | Clinician |
| 1. Annika Volke | Clinician |
| 1. Ashish Bansal | Pharmaceutical Industry |
| 1. Beth Stuart | Methodologist |
| 1. Bo Bang | Pharmaceutical Industry |
| 1. Brian Calimlim | Pharmaceutical Industry |
| 1. Carl-Fredrik Wahlgren | Clinician |
| 1. Chanho Na | Clinician |
| 1. Christian Apfelbacher | Methodologist |
| 1. Claire Feeney | Pharmaceutical Industry |
| 1. Dong Hun Lee | Clinician |
| 1. Dora Stölzl | Clinician |
| 1. Eri Maruyama | Patient / patient representative |
| 1. Eric Simpson | Clinician |
| 1. Fabio Nunes | Pharmaceutical Industry |
| 1. Henrique Ishii | Patient / patient representative |
| 1. Henrique Teixeira | Pharmaceutical Industry |
| 1. Hidehisa Saeki | Clinician |
| 1. Hiroyuki Murota | Clinician |
| 1. Hiroyuki Toyama | Pharmaceutical Industry |
| 1. Hyejung Jung | Clinician |
| 1. Hywel Williams | Clinician |
| 1. Isabelle Guillemin | Pharmaceutical Industry |
| 1. Jan Gutermuth | Clinician |
| 1. Jean-francois stalder | Clinician |
| 1. Jennifer Austin | Patient / patient representative |
| 1. Jiyoung Ahn | Clinician |
| 1. Joanne Chalmers | Methodologist |
| 1. Jochen Schmitt | Clinician |
| 1. Jooyoon Bae | Clinician |
| 1. Julie Block | Patient / patient representative |
| 1. Katrina Abuabara | Clinician |
| 1. Kazue Yoshida | Clinician |
| 1. Ken Igawa | Clinician |
| 1. Kim Thomas | Methodologist |
| 1. Kyoko Maru | Patient / patient representative |
| 1. Louise Gerbens | Clinician |
| 1. Laura Howells | Methodologist |
| 1. Laura von Kobyletzki | Clinician |
| 1. Laurent Eckert | Clinician |
| 1. Linda Wang | Pharmaceutical Industry |
| 1. Lynita Howie | Patient / patient representative |
| 1. Magdalene Dohil | Clinician |
| 1. Mami Murakami | Pharmaceutical Industry |
| 1. Maria Bradley | Clinician |
| 1. Masaki Futamura | Clinician |
| 1. Masutaka Furue | Clinician |
| 1. Michaela Gabes | Methodologist |
| 1. Michael Lanigan | Patient / patient representative |
| 1. Miwako Ogino | Patient / patient representative |
| 1. Norito Katoh | Clinician |
| 1. Phyllis Spuls | Clinician |
| 1. Rai Fujimoto | Clinician |
| 1. Rosemary Humphreys | Patient / patient representative |
| 1. Sebastien Barbarot | Clinician |
| 1. Stephan Weidinger | Clinician |
| 1. Susumu Ichiyama | Clinician |
| 1. Tae Young Han | Clinician |
| 1. Takeshi Nakahara | Clinician |
| 1. Tatsuki Fukuie | Clinician |
| 1. Teresa Berents | Clinician |
| 1. Tim Burton | Patient / patient representative |
| 1. Toshiya Ebata | Clinician |
| 1. Vanessa Sultana | Patient / patient representative |
| 1. Yasutomo Imai | Clinician |
| 1. Yik Weng Yew | Clinician |
| 1. Yoko Kataoka | Clinician |
| 1. Yukihiro Ohya | Clinician |
| 1. Yuko Ikegami | Patient / patient representative |
